# The use of a simple and affordable skin patch for measurement of transcutaneous bilirubin levels in neonates during phototherapy

**DOI:** 10.3389/fped.2024.1434770

**Published:** 2024-09-25

**Authors:** Aditya Kallimath, Suprabha Patnaik, Pradeep Suryawanshi, Rupeshkumar Deshmukh, Nandini Malshe

**Affiliations:** ^1^ Department of Neonatology, Bharati Vidyapeeth Deemed University Medical College and Hospital, Pune, India; ^2^ Department of Community Medicine, Bharati Vidyapeeth Deemed University Medical College and Hospital, Pune, India

**Keywords:** hyperbilirubinemia, neonate, phototherapy, skin patch, transcutaneous bilirubin

## Abstract

**Background:**

Transcutaneous bilirubin (TcB) measurements during and after phototherapy for hyperbilirubinemia must be performed on unexposed skin. There are commercially made skin patches for this purpose, but they are relatively unavailable in low-resource settings. We devised a simple cotton patch and tested its use for TcB during phototherapy.

**Methods:**

Measurements were taken in healthy neonates born at a gestational age of ≥35 weeks who were undergoing phototherapy for hyperbilirubinemia in western India before, 12 h after the start, and 12 h after the end of phototherapy. Total serum bilirubin (TSB) was measured using the diazo method in a clinical laboratory. TcB measurements were performed using a Dräger Jaundice Meter JM-105 placed over the sternum on two skin areas that were protected during and after treatment by a commercial (Philips BilEclipse) or self-made patch comprised of cotton gauze and wool.

**Results:**

In total, 47 neonates were included in our study. Before phototherapy, TSB and TcB values had a strong correlation (Pearson, *r* = 0.88), with a mean difference of −1.35 mg/dl. Correlations with TSB were good and equivalent for TcB values measured on skin covered by the commercial and self-made patches during (0.78 and 0.70, respectively) and after (0.57 and 0.58, respectively) phototherapy. TcB values measured on skin covered by the two patches correlated well both during and after phototherapy, with *r* = 0.82 and 0.90, respectively, and mean (95% confidence interval) differences of −1.21 and −0.32 mg/dl, respectively.

**Conclusions:**

Reliable TcB measurements taken during and after phototherapy can be achieved on skin covered with a simple and affordable cotton skin patch.

## Introduction

It is estimated that hyperbilirubinemia, sometimes observed as visible jaundice, occurs in approximately 80% of all neonates (1). The traditional method of measuring bilirubin levels in the blood requires an invasive and painful procedure of collecting blood by pricking a peripheral vein of the neonate and requires a clinical laboratory. Alternatively, bilirubin levels can be estimated non-invasively and immediately at the bedside using transcutaneous bilirubinometers.

While these handheld instruments have varying sensitivities and accuracies, transcutaneous bilirubin (TcB) levels have been shown to closely correlate with total serum bilirubin (TSB) measurements. For example, Kurnianto et al. found a good correlation between TSB and TcB values using the Dräger Jaundice Meter JM-103 (Lubeck, Germany) instrument placed on the forehead or sternum of neonates born at a gestational age (GA) of >38 weeks (2). TcB measurement is not advised in neonates after initiation of phototherapy because phototherapy can bleach the skin and thereby affect measurement accuracy (3, 4). A solution for this is to measure TcB on a patch of skin that has been covered during phototherapy.

Using the Philips BiliCheck (Andover, Massachusetts, USA) transcutaneous bilirubinometer on skin covered with a Philips BilEclipse (Andover, Massachusetts, USA) phototherapy patch, Alsaedi found a good correlation between TSB and TcB values during phototherapy of full-term neonates (5). As commercially available patches such as the BilEclipse are unavailable, cost prohibitive, or otherwise a burden on resources in most of the world, we created a simple patch from readily available medical cotton and tested its utility for performing TcB measurements during phototherapy.

## Material and methods

### Study design

**Figure 1:**
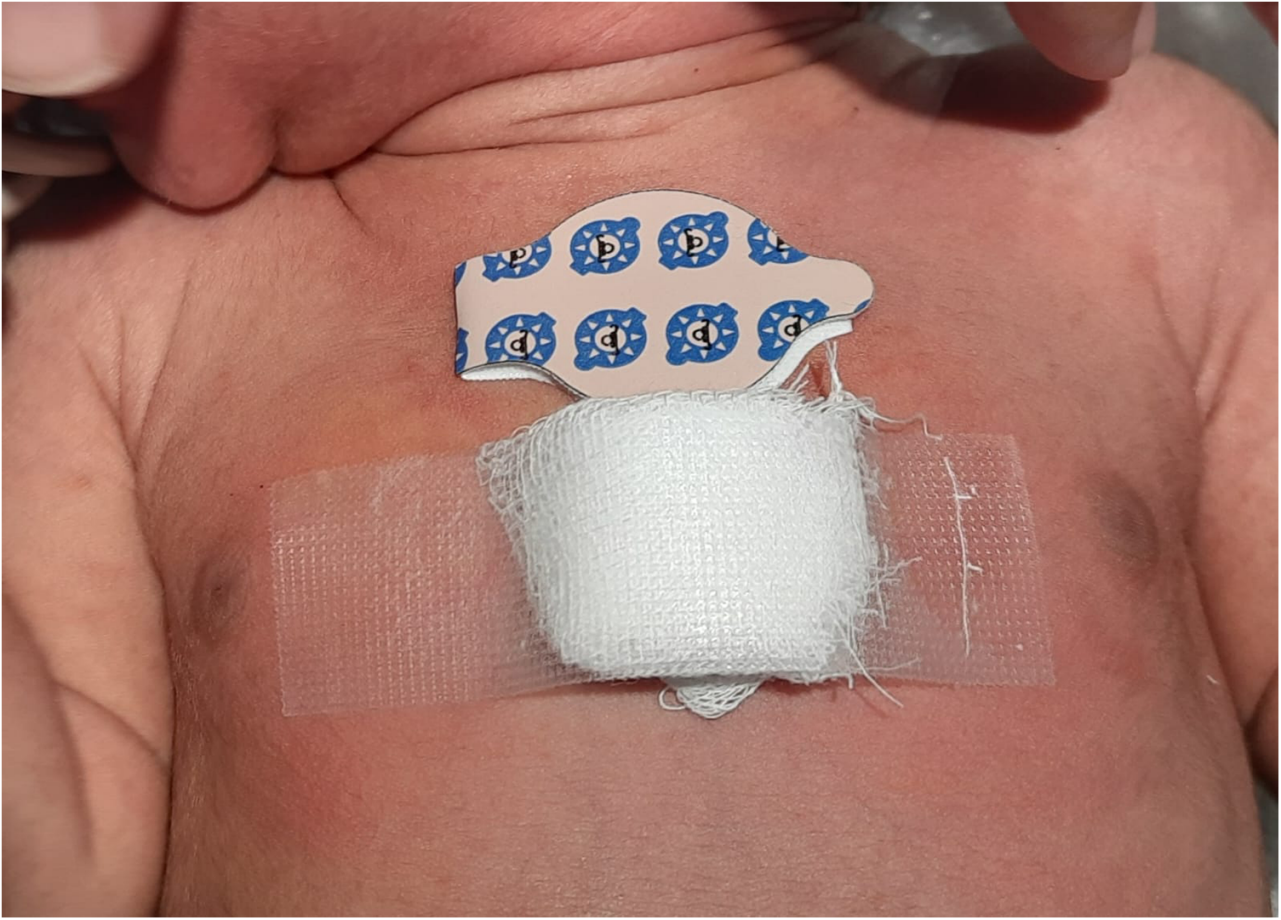
Photograph of a neonate with the commercial BilEclipse and the self-made skin patches on the sternum. The self-made patch is made of cotton and affixed with medical adhesive tape.

This prospective cohort study was conducted from October 2020 to May 2021 in the neonatal intensive care unit (NICU) of a tertiary care hospital affiliated with a medical college in western Maharashtra. Ethics committee approval was obtained before starting the study (identifier BVDUMC/IEC/26). Neonates with a GA >35 weeks admitted to the NICU without a history of phototherapy were screened. Those who were clinically unstable for any reason, such as having sepsis, poor perfusion for any reason, hydrops fetalis, skin bruising, ecchymosis, hyperpigmentation or hemangioma over the sternal area, conjugated hyperbilirubinemia, or major congenital anomalies, were excluded. Written informed consent was obtained from the parents. The neonates were clinically evaluated for jaundice and their TcB levels were measured using a Dräger Jaundice Meter JM-105. If the level indicated the need to initiate phototherapy as per the 2004 American Academy of Pediatrics (AAP) clinical practice guidelines (6), a venous blood sample was then collected within 15 min for measurement of TSB levels using the diazo method and Abbott Alinity Ci series equipment (Lake county, Illinois, USA). Phototherapy was started within the next 1 h and was administered on a Zeal Medical LED Phototherapy Unit (Mumbai, India) series 4100 unit at 25–30 µW/cm^2^/nm in the 430–490 nm band. The neonates wore locally made eye goggles and their genitals were covered with a nappy. Phototherapy lasted an average of 12–16 h. Two types of skin patches were applied over the upper sternum before the phototherapy started. One was an adhesive, 2.5-cm diameter Philips BilEclipse phototherapy protective patch that was applied over the upper sternum and is referred to as patch A. The other was a patch that we designed and applied over the central sternum and is referred to as patch B. This self-made patch was made at the bedside by sandwiching a 3–4-mm thick layer of medical cotton wool between two 2.5-cm square, 8-ply cotton gauze pieces (Figure 1) and secured over the skin with adhesive medical tape. The BilEclipse patch has a re-sealable flap that was opened to uncover skin for TcB measurement. With our patch, its adhesive tape can be partially lifted to uncover skin for TcB measurement. TSB and TcB were measured during phototherapy (12 h after starting) and 12 h after the end of phototherapy. Blood sampling for TSB was conducted within 15 min of a TcB measurement. Phototherapy was turned off during each TcB measurement and blood sampling. Clinical data such as GA, mode of delivery, birth weight, postnatal age, and etiology of jaundice were collected from a chart review.

### Statistical analyses

The sample size was calculated based on previous studies that reported correlation coefficients of 0.3–0.6 between post-phototherapy TSB and TcB measurements. For a correlation coefficient of 0.40, a sample size of 47 allowed for a power of 0.80 at alpha = 0.05. Paired *t*, Pearson correlation, and Bland–Altman analyses were performed using IBM SPSS software (version 28), and a *p*-value <0.05 was deemed significant.

## Results

### Study population

The TSB and TcB levels in 47 neonates (26 females) with a GA of ≥35 weeks were measured before, during, and after phototherapy (Table 1). The mean (SD) GA and birth weight were 37.7 (1.4) weeks (1.4) and 2,747 (404) g, respectively. TcB measurements during and 12 h after the end of phototherapy were taken from two areas of the sternum that had been kept covered by either a commercial (BilEclipse) or a self-made patch (Table 1).

**Table 1:** Levels of bilirubin of 47 neonates with hyperbilirubinemia before, during, and after phototherapy.

	TSB (mg/dl), range; mean (SD)	TcB(mg/dl), range; mean (SD)	TcB–TSB difference, mean (SD); 95% confidence interval
Before	4.10–25.70; 14.57 (4.71)	2.30–22.00; 13.22 (3.92)	−1.35 (2.19); −5.64 to 2.94
During	6.30–20.00; 10.36 (3.44)	A: 1.30–19.50; 6.92 (4.01) B: 1.50–19.80; 8.48 (4.02)	A: −3.44 (2.52); −4.18 to 2.70; B: −1.88 (2.92); −7.60 to 3.84
After	5.70–14.90; 10.30 (2.28)	A: 3.40–14.10; 8.76 (2.63) B: 3.40–13.70; 9.08 (2.62)	A: −1.53 (2.27); −5.99 to 2.92 B: −1.21 (2.25); −5.62 to 3.20

Measurements were obtained 15 min prior, 12 h into, and 12 h after phototherapy sessions of 12–16 h. TcB was measured on the sternum which was covered by a commercial (A) or a self-made (B) patch.

### Bilirubin values before the start of phototherapy

**Figure 2:**
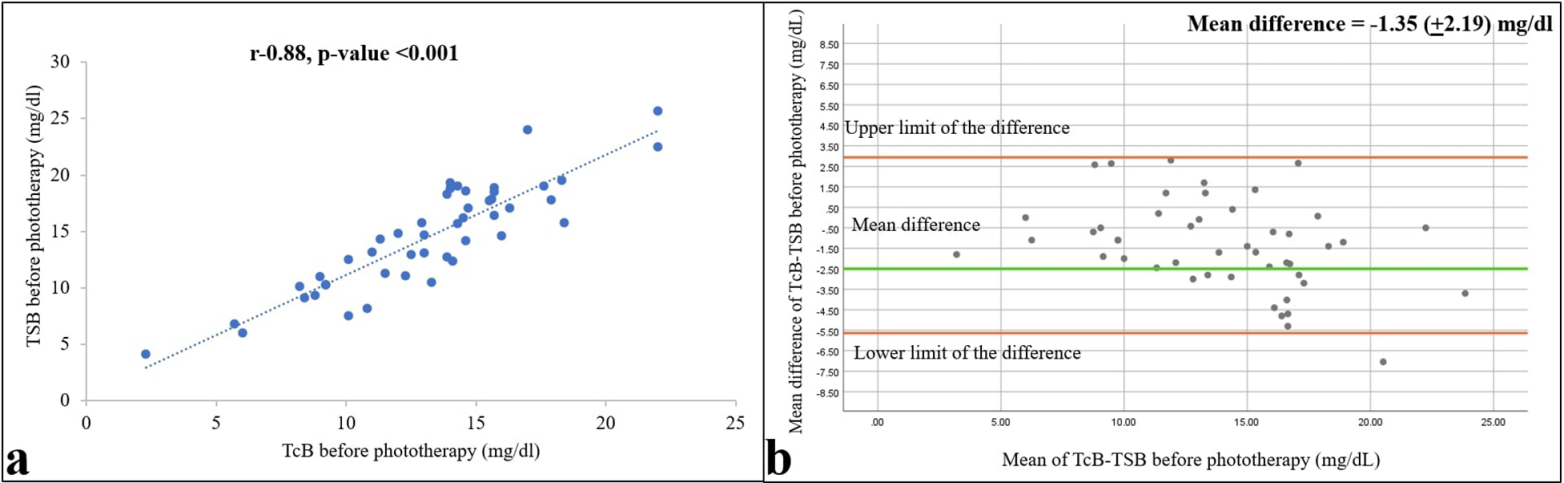
Relationships between TSB and TcB values within 1 h before starting phototherapy are shown as a correlation **(a)** and Bland–Altman **(b)** plots. Pearson correlation coefficient and *p*-value, and 95% confidence limits and mean differences are shown (*n* = 47).

Mean (SD) TSB and TcB values before the start of phototherapy were 14.57 (4.71) and 13.22 (3.92) mg/dl, respectively (1 mg/dl = 17.1 µM). The two measurements correlated strongly, with a Pearson *r* = 0.88 (*p* < 0.001), and differed by −1.35 mg/dl [95% confidence interval (CI) = −1.99 to −0.07; Figure 2].

### Bilirubin values during phototherapy and after the end of phototherapy

**Figure 3:**
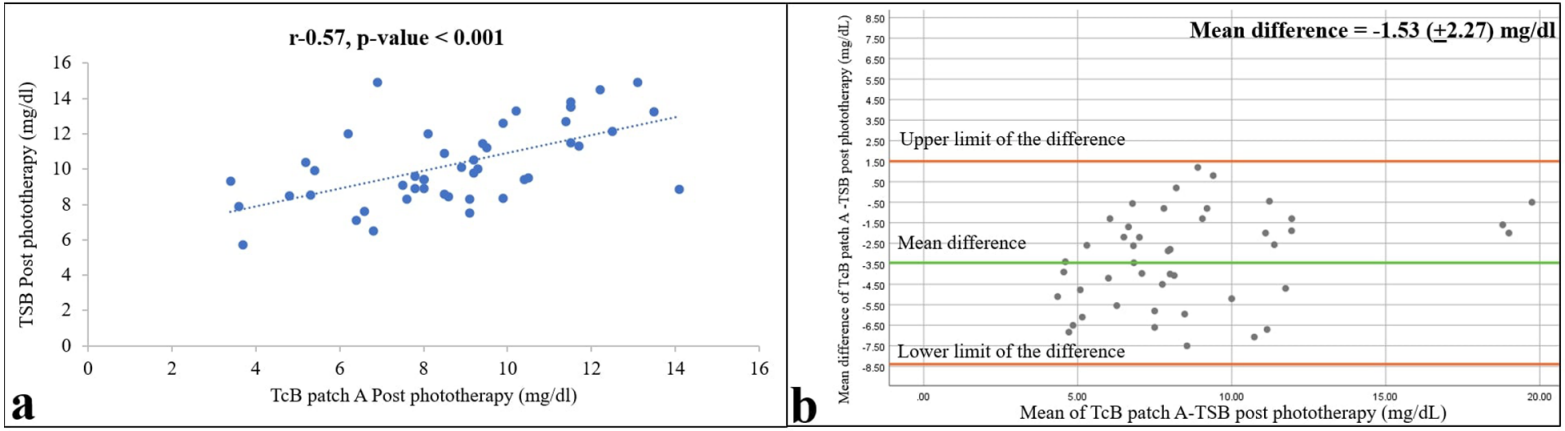
Relationships between TSB and TcB values on skin areas covered with the commercial patch (patch A) 12 h after the end of phototherapy are shown as a correlation **(a)** and Bland–Altman **(b)** plots. Pearson correlation coefficient and *p*-value, and 95% confidence limits and mean differences are shown (*n* = 47).

**Figure 4:**
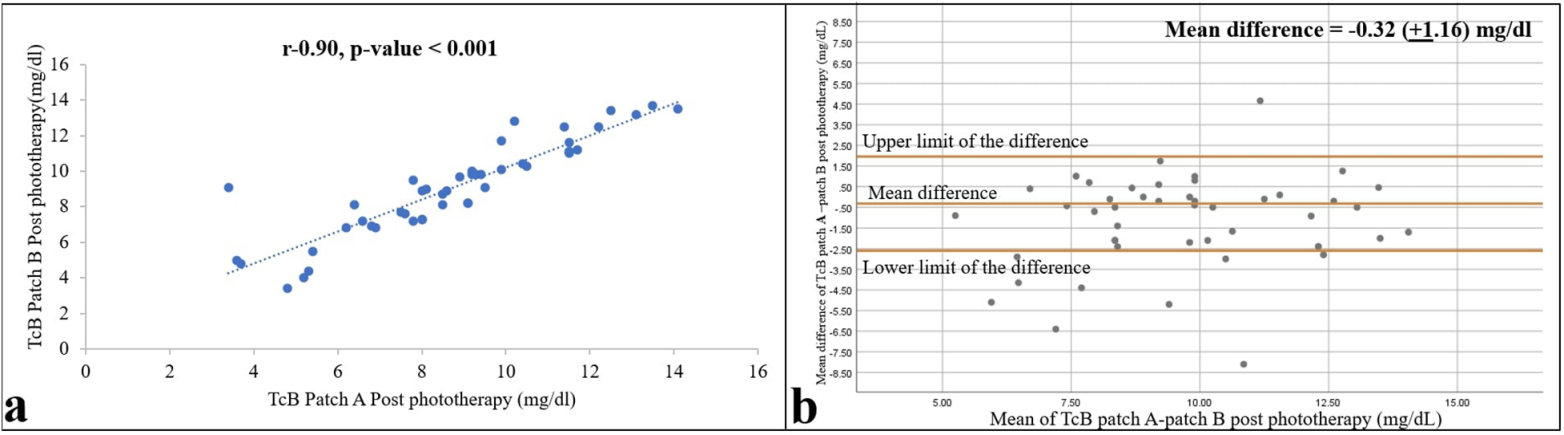
Relationships between TSB and TcB values on skin areas protected by the self-made patch (patch B) 12 h after the end of phototherapy are shown as a correlation **(a)** and Bland–Altman **(b)** plots. Pearson correlation coefficient and *p*-value, and 95% confidence limits and mean differences are shown (*n* = 47).

As expected, bilirubin levels in the bodies of the neonates were reduced by phototherapy. The mean (SD) TSB and TcB values under the commercial and self-made patches were 10.36 (3.44), 6.92 (4.01), and 8.48 (4.02) mg/dl, respectively, 12 h after the start of phototherapy, and 10.30 (2.28), 8.76 (2.63), and 9.08 (2.62) mg/dl, respectively, 12 h after the end of phototherapy. However, compared with the values before phototherapy, concordance between the TSB and TcB measurements was lower during and after phototherapy, with Pearson *r* values of 0.78 and 0.70 during phototherapy and 0.57 and 0.58 after phototherapy (Figures 3, 4) for the commercial and self-made patches, respectively (all *p*-values < 0.001).

### Comparison of the TcB measurements under the two patches

**Figure 5:**
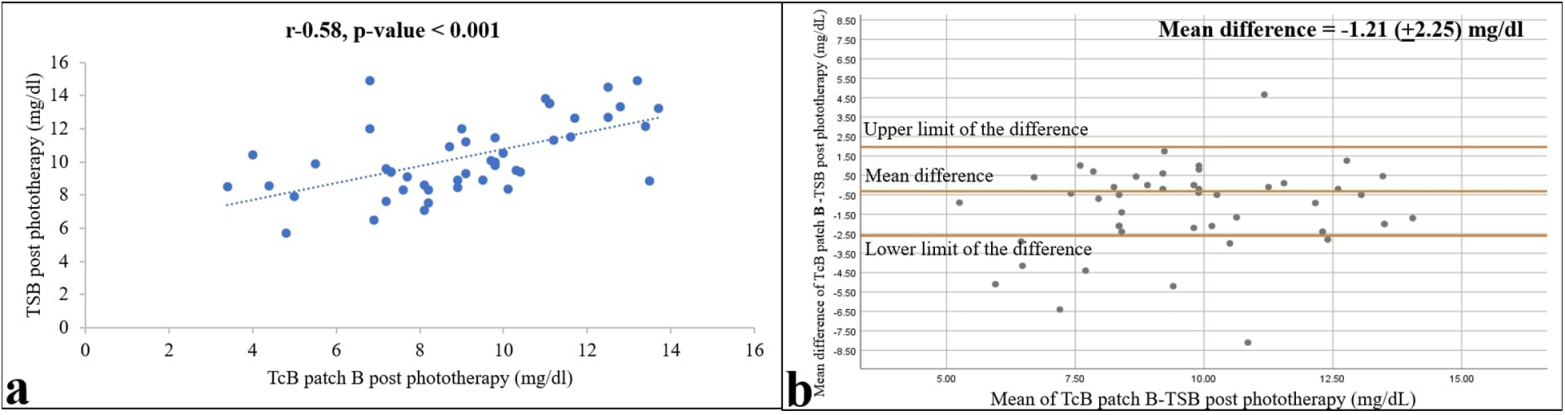
Relationship of TcB values from skin areas covered with a commercial (patch A) and self-made (patch B) patch 12 h after ending phototherapy is shown as a correlation **(a)** and Bland–Altman **(b)** plots. Pearson correlation coefficient and *p*-value, and 95% confidence limits and mean differences are shown (*n* = 47).

The TcB measurements under the two types of patches correlated well both during and after phototherapy, with *r* = 0.82 and 0.90 (both *p*-values < 0.001), and mean (95% CI) differences of −1.21 (−1.87 to −0.55) and −0.32 (−0.06 to –0.02), respectively (Figure 5). Paired *t*-tests revealed a significant difference between the two patches during phototherapy (*p* = 0.001), whereas the TcB measurements after phototherapy showed no difference (*p* = 0.06).

## Discussion

Several studies on preterm and term infants have noted that covering the skin with the BilEclipse patch allows for reliable TcB measurements, with a good correlation observed between the TSB and TcB levels during and after phototherapy (7–11). We too observed good concordance between the TSB and TcB values using the BilEclipse patch in our study. The darker skin tone of Indian infants in our cohort did not significantly affect the reliability of the TcB measurements. However, a study of neonates performed in central India by Murli et al. did not find a satisfactory correlation between TSB and TcB values before, during, or after phototherapy (12). This may be attributable to the relatively wide range of TSB (mean −17.2 mg/dl) in the study cohort for which the performance of the BiliCheck tool, which the authors used, has been found to be inaccurate (13).

While our study supported what many other studies (14, 15) have demonstrated (that TcB devices are reliable tools for screening), we also showed that skin coverage during and after phototherapy is easily achieved with simple cotton-made patches assembled at the bedside and allows for reliable TcB measurements. TcB levels have to be determined on skin areas that are covered during phototherapy because exposure to light bleaches the skin. Commercially available skin patches are available for this purpose, such as the BilEclipse patch, but are costly for low-resource settings, especially in low- and middle-income countries such as India, and are also often unavailable. To this end, we designed an alternative skin patch using cotton gauze, cotton wool, and adhesive tape and found that it was not inferior to the BilEclipse patch for reliable TcB measurements taken during and after phototherapy. Other alternatives to commercial phototherapy-shielding patches have been evaluated. Costa-Posada et al. showed that a commercial patch to shield skin for thermometry can be used as a skin patch for reliable TcB measurements in both term and preterm neonates (16). Pal et al. used a disposable electrocardiogram electrode patch 2.5cm in size covered with aluminum foil as a patch for TcB measurements, which corresponded well with TSB measurement during phototherapy (17). However, unlike in these two studies, our self-made patch is not a repurposed commercially manufactured product. Instead, it is assembled at the bedside using cotton material that is available in any medical setting. Although paired *t*-tests revealed a significant difference between the two patches used during phototherapy, there was a good correlation between the TcB measurements of the two patches and also a good correlation between the TSB values. The TcB measurements showed a good correlation between the two patches after phototherapy and also had no significant difference. Our self-made patch was not assessed for light-impermeability using an illuminance meter, unlike the commercially available patch that has undergone testing and validation. There could have been light passing through the self-made patch during phototherapy, which could account for the discrepancy between the TcB measurements using the two patches during phototherapy.

In our study, we measured TSB and TcB 12 h after the start of phototherapy. The most significant decline in TSB usually occurs 6 h after the initiation of phototherapy. Phototherapy can decrease TSB by 20%–30% in the first 12–24 h (18). The recent clinical practical guideline revision published by the AAP in the management of hyperbilirubinemia in neonates with a gestational age of >35 weeks also recommends testing for bilirubin levels 12 h after the start of phototherapy (1). In general, both intravascular (found within capillaries and arterioles) and extravascular tissue spaces make up the skin volume that TcB devices measure. The bilirubinometers adjust for the variations in concentration between the intravascular and extravascular spaces using internal calibration parameters (19). Because TSB equals the bilirubin concentration in the intravascular space and TcB primarily includes extravascular bilirubin contribution, the two parameters are physiologically distinct from one another. The relationship between TSB and TcB is dependent on several factors that regulate the extravasation and clearance of bilirubin in the extravascular space, such as the photolysis of bilirubin by phototherapy or daylight influences, or are still poorly understood, such as the cephalocaudal progression of jaundice, even though many studies, including our own, have demonstrated a strong correlation between TSB and TcB (20, 21).

Phototherapy acts on circulating bilirubin molecules in the dermal capillaries and the principal mechanism by which phototherapy works is by converting bilirubin into lumirubin (structural isomerization), which is more soluble and non-neurotoxic than bilirubin, hence lowering bilirubin levels. Bile and urine then excrete lumirubin without conjugation. As bilirubin travels through the skin's superficial capillaries, direct exposure to photons released by the light source results in the conversion. A minor amount of bilirubin removal may also be explained by photo-oxidation to polar molecules and photoisomerization to the less hazardous 4Z, 15Z bilirubin isomer (22). The excretion half-lives of the byproducts of configurational isomerization (bilirubin isomer 4Z, 15E) and structural isomerization (lumirubin) are 13 and 1.9 h, respectively. While structural isomerization is not reversible, configurational isomerization is, and it occurs considerably more quickly. Structural isomers do not significantly accumulate in the blood since they are excreted so quickly. Conversely, the configurational isomers are eliminated at a far slower pace and have been shown to accumulate to an apparent photostationary level of approximately 25% of TSB when exposed to phototherapy (23–25). Hence, there is always a possibility of a rebound bilirubin increase and we usually test for the same 12 h after stopping phototherapy as recommended by recent clinical guidelines (1).

This study provided a novel and practical approach for TcB monitoring in resource-limited settings. However, the self-made patch was not tested for light-impermeability using an illuminance meter. Although our study showed a correlation between the self-made patch and the commercially available patch with regard to TcB measurements, this requires further scientific validation. Larger studies are required before the self-made patch for TcB monitoring can be recommended. It should also be noted that our study was conducted at a single center in western India and the observed value of the self-made patch may not be applicable to neonates of other Asian or Western ethnicities. Our study excluded preterm neonates born at a GA of <35 weeks as well as sick neonates.

## Conclusion

The use of TcB devices in neonates during and after phototherapy for hyperbilirubinemia is a reliable screening method when measurements are performed on skin that is covered from exposure to light treatment. While commercial patches such as BilEclipse are made for this purpose, we provide evidence for a more practical, simple, and affordable skin patch that can be readily assembled at the bedside.

## Data Availability

The raw data supporting the conclusions of this article will be made available by the authors, without undue reservation.
